# Effect of Processing Conditions on the Flash Onset Temperature in Hydroxyapatite

**DOI:** 10.3390/ma14185229

**Published:** 2021-09-11

**Authors:** Changhun Hwang, Jondo Yun

**Affiliations:** 1 Department of Advanced Engineering, Graduate School, Kyungnam University, Changwon 51767, Korea; hchandpsy@naver.com; 2 Department of Advanced Materials Engineering, Kyungnam University, Changwon 51767, Korea

**Keywords:** flash sintering, hydroxyapatite, flash onset temperature, fast sintering

## Abstract

When heat and electric field are applied to the sample, sintering takes place within a short time of a few seconds by the flash phenomenon that occurs. In what condition flash does occur is a main issue for the flash sintering technique. In this study, the effect of processing conditions such as sintering atmosphere, sample size, density and grain size on the flash onset of hydroxyapatite was investigated. In a vacuum atmosphere, a flash occurred at a lower temperature by 50–100 °C than in air. The smaller the thickness of the sample, the higher the flash onset temperature due to the larger specific surface area. Flash was also observed in samples which were presintered, having a density of 86–100% and a grain size of 0.2–0.9 μm. When the density and grain size of the sample were higher and larger, the flash onset temperature was higher. It was because the diffusion and conduction path through the grain boundary and the inner surface of the pores with high defect concentration are blocked with an increase of density or grain size. When an electric field was applied during flash sintering, a color change of the sample was observed and the reason was discussed.

## 1. Introduction

Flash sintering is a process of consolidating loose powders using thermal energy with an application of electric field. It is similar to or belongs to field assisted sintering technique (FAST) or electric field assisted sintering (EFAS) in the sense of using electric field [1]. It is possible to produce a dense body at relatively lower temperatures in a short time within seconds by applying an electric field to the sample, causing a flash phenomenon [2,3]. When flash occurs, the sample temperature rises by several hundred degrees instantaneously and densification occurs abruptly. It is interesting for one to find that the flash occurs at a specific temperature given by the processing conditions [4,5,6,7,8,9,10,11,12,13,14]. Zhang et al. [15] stated that the flash sintering temperature of the zinc oxide sample in a reducing atmosphere was 350~400 °C lower than in air. Biesuz et al. [16] reported that the electrical conductivity increased when argon gas was sprayed on 8 mol% yttria-stabilized zirconia (8YSZ) sample during flash onset. Francis et al. [17] reported that the larger the particle size of 3 mol% yttria-stabilized zirconia (3YSZ) raw material powders, the higher the flash onset temperature and the lower the densification rate. Avila et al. [18] reported that the thinner the thickness of the 3YSZ sample, the lower the densification rate during flash sintering, which was due to the specific surface area of the sample.

The hydroxyapatite ceramic is manufactured by using various sintering techniques. In case of normal sintering, a dense body can be obtained by sintering under predetermined conditions at temperatures from 1250 °C or higher for several hours [19]. However, if the sintering time and temperature are over the range of proper condition, thermal decomposition, grain growth, or phase change may occur leading to deterioration of properties [20,21]. Spark plasma sintering which is a kind of electric field-assisted sintering was also used to effectively produce a dense body of hydroxyapatite [22,23,24], even though it is costly by using a dedicated mold and high pressure. Recently, flash sintering method was introduced to successfully manufacture hydroxyapatite without thermal decomposition and phase change at a low temperature for a short time within seconds [25,26]. However, temperature and voltage conditions for flash onset were not fully investigated especially with a variable of the sample and processing conditions.

In this study, the flash onset temperature of hydroxyapatite was investigated by changing variables such as gas atmosphere, sample size, and presintering temperature/time under the flash conditions.

## 2. Materials and Methods

Hydroxyapatite powders (Junsei, Tokyo, Japan) were uniaxially pressed to prepare a thin rectangular plate-shaped green sample, with a size of 20 × 5.4 × 1 mm, which was again pressed by cold isostatic pressing (CIP-L2-70-200, Suflux, Deajeon, Korea) for 5 min under a pressure of 200 MPa. Two holes having a diameter of 1 mm were drilled onto the pressed green sample in a thickness direction at an interval of 10 mm in the longitudinal direction. The sample was hung by a platinum wire hooked in two holes and a platinum paste was applied to the holes to lower the contact resistance.

Flash sintering was carried out under two conditions: An elevating temperature condition and an isothermal condition. In the case of elevating temperature condition, the sample was placed in a furnace and heated with a rate of 10 °C/min. When 800 °C was reached, an electric field was applied and, during temperature rising, the onset temperature of flash was recorded. In the case of isothermal condition, the sample was heated to the desired temperature and an electric field was applied to generate flash. Before flash sintering, some samples were presintered by normal sintering method without an application of electric field at 1000, 1100, or 1200 °C for 5, 60, 120, 180, or 240 min respectively.

DC power supply (XR Series 2000V/1A, Magna Power, Flemington, NJ, USA) was used for the power source. As the current surges when flash occurs, voltage control was automatically converted to current control and current limiting values of 3 mA in air and 100 mA in vacuum were used. During the entire flash sintering experiment, voltage, current, and surface temperature of the sample were recorded using a digital multimeter and pyrometer, and the morphology of the sample was recorded using CCD (IS 6 Advanced, Lumasense, Ballerup, Denmark).

The flash-sintered portion between the positive and the negative electrodes was selectively cut and collected, and its density was measured by the Archimedes method. After polishing to about 50% of the thickness of the sample, thermal etching was done for 10 min in the 900 °C, and microstructure analysis was performed with a scanning electron microscopy (Merlin compact, Zeiss, Germany). From the obtained microstructure image, the average grain size was measured using the linear intercept method. Phase analysis was performed using an x-ray diffractometer (D/Max-2500VL/PC, Rigaku, Tokyo, Japan).

## 3. Results and Discussion

**Figure 1:**
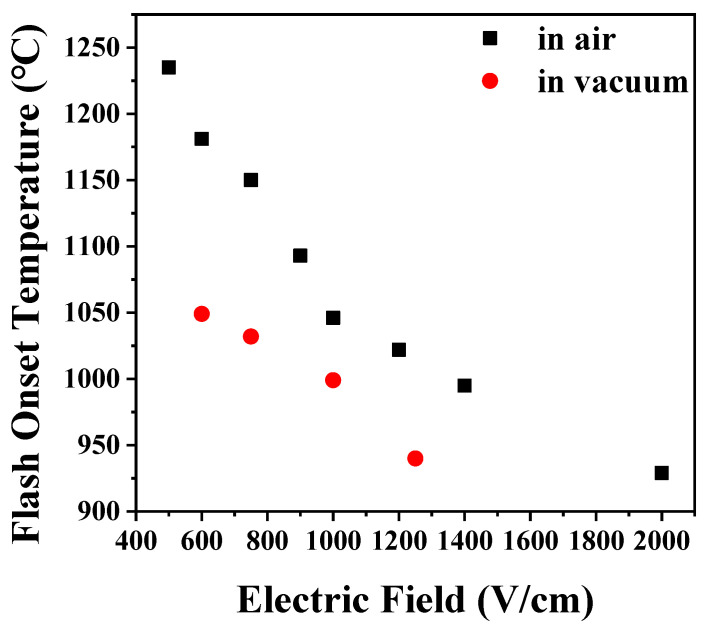
Change of flash onset temperature with electric field during flash sintering of hydroxyapatite.

**Figure 2:**
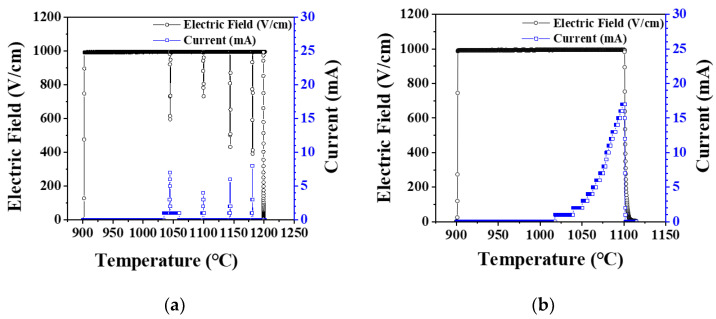
Electric field and current measured during flash sintering of hydroxyapatite under elevating temperature condition (**a**) in air and (**b**) in vacuum.

**Figure 3:**
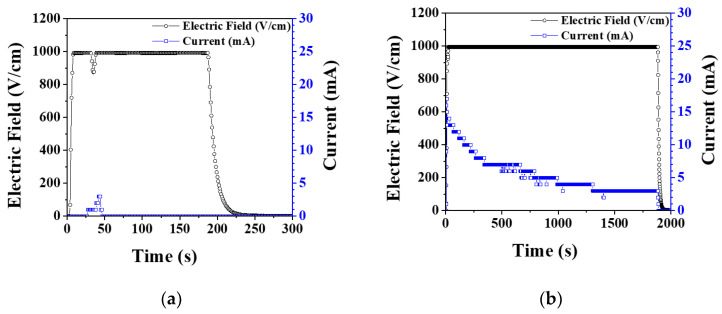
Electric field and current measured during flash sintering of hydroxyapatite under isothermal conditions at 1050 °C and 1000 V (**a**) in air and (**b**) in vacuum.

Under elevating temperature condition, the flash onset temperature decreased as the electric field increased, and it was lower in vacuum by 50–100 °C than in air, as shown in Figure 1. In air, when flash occurred, the current rapidly increased, and as the temperature was elevated, several more flashes occurred, as shown in Figure 2a. On the other hand, in vacuum, the current slowly increased as the temperature increased, as shown in Figure 2b, and the flash occurred only once. Under an isothermal condition of 1050 °C with applied 1000 V, the flash occurred only once in air. In vacuum, as in the case of the elevating temperature condition, a flash occurred once, and the current was found to change slowly (Figure 3).

As hydroxyapatite materials generate hydroxyl and hydrogen defects by temperature-dependent dehydration reaction, electrical conductivity increases [27,28,29]. In a vacuum atmosphere, dehydration reaction is accelerated, defect concentration increases, and flash occurs at lower temperatures [30,31,32,33,34]. In the elevating temperature condition, since the defect concentration increases as the temperature rises, multiple flashes may occur, However, in the isothermal condition, the defect concentration is constant, so multiple flashes may not occur. In vacuum, the current abruptly rose and then slowly decreased. As mentioned later in this paper, the reason for the decrease in current in vacuum is that grain growth and densification proceed over time, so the grain boundary fraction decreases and resistance increases.

**Figure 4:**
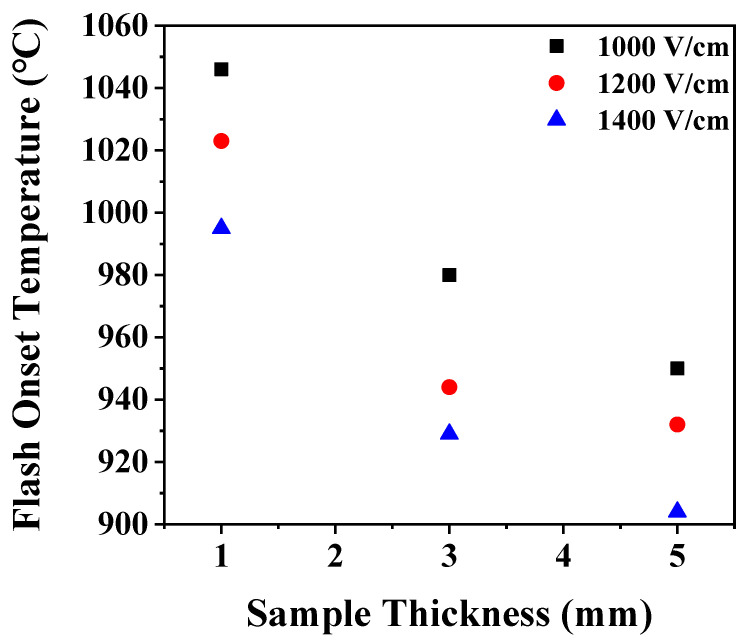
Change of flash onset temperature with a sample thickness.

Flash onset temperature was found to be a function of the thickness of the samples. It decreased as the thickness increased (Figure 4). Since sample has a heat source inside during flash sintering, the internal temperature of the sample is higher than the furnace temperature making heat loss through the surface and thus a size effect. When the sample size increases, the rate of increase in surface area is smaller than that in volume, so the specific surface area decreases. When the thickness increases by three or five times, the specific surface area decreases by 46% or 57%, respectively. The reduction of the specific surface area causes a reduction in heat loss and preservation of heat in the samples, thereby lowering the flash onset temperature.

**Figure 5:**
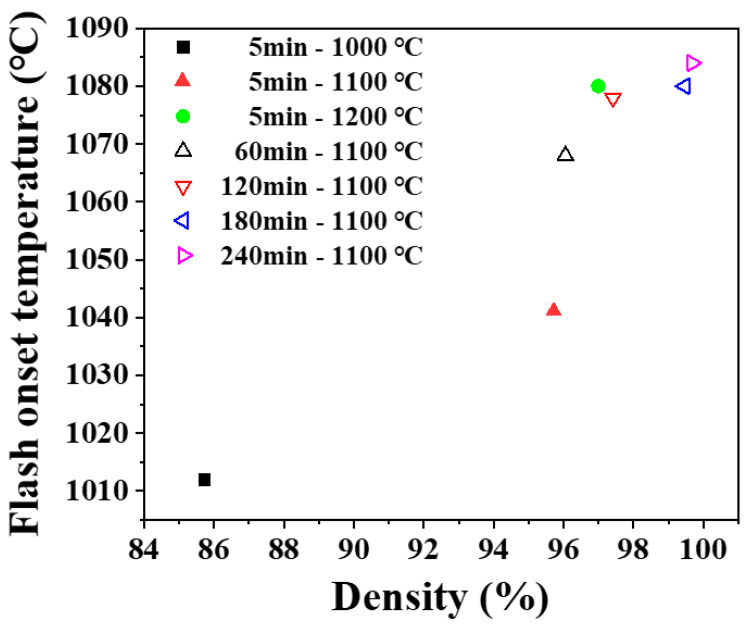
Change of flash onset temperature with a density of presintered sample. Legend shows presintering condition.

**Figure 6:**
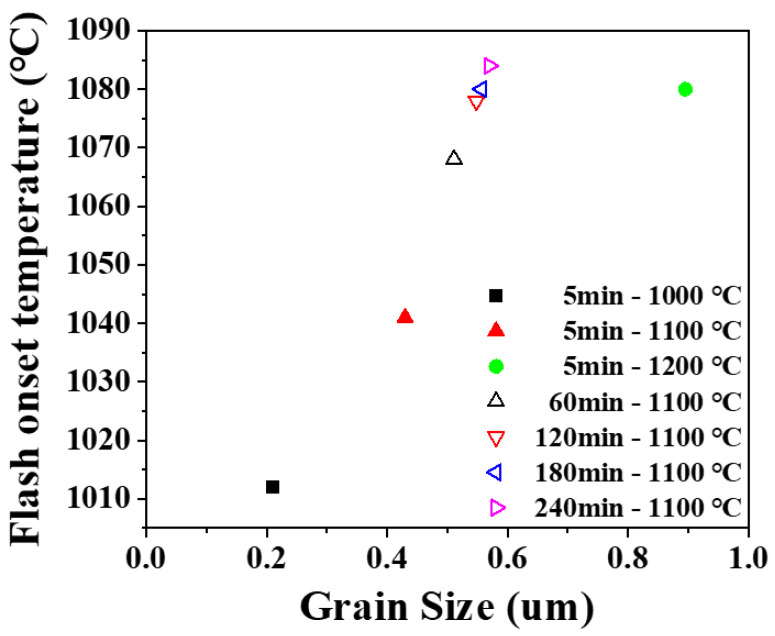
Change in flash onset temperature with a grain size of presintered sample. Legend shows presintering condition.

Flash sintering was performed on the presintered samples to investigate the effect of microstructure. After normal sintering, presintered samples had densities in a range of 86% to 100% and grain sizes in a range of 0.2 to 0.9 μm. Flashes were observed in all samples with high or full density, indicating that flash does occur not only in the sintering process of loose powder but also in the dense body. Flash onset temperature was found a function of density and grain size as shown in Figure 5 and Figure 6. Onset temperature was higher when the density and grain size larger. In ceramics, the electrical conduction is through atomic diffusion by defects. It is well known that diffusion through the surface or grain boundary is faster than through lattice. When the density is low and the porosity is high, the surface area inside the pores is large, and electrical conduction becomes easier. [35,36,37] When the grain size is smaller, the grain boundary area is larger, and the more favorable the flash onset [17].

If the densification and grain growth proceeds during flash sintering, the temperature required for the flash onset will increase. Under isothermal conditions, flash can be suppressed once it has occurred. However, under the elevating temperature condition, the flash may occur again when temperature becomes sufficiently high enough to meet the required defect concentration.

When the hydroxyapatite green body was normal sintered in air, the color of the sample changed from white to pale blue. The color became darker as the sintering temperature was higher. When the sample with a pale blue color was flash sintered again, the color disappeared and the sample was whitened in the portion between the negative and the positive electrodes. The whitening occurred only in the inner part in between two electrode holes where there is an influence of the electric field, indicating that it is an effect of the electric field.

Changes in color during sintering of hydroxyapatite have been reported [38,39,40,41]. Bystrov et al. [40] noticed a change in the bandgap when sintering hydroxyapatite and a specific visible light is absorbed to give it a blue color. This is due to the oxygen vacancies in the OH and PO_4_ group, and they reported that the color depth varies with the amount of oxygen vacancies formed. Yubao et al. [41] reported that oxidation of the manganese impurity made a change to blue color. However, quantitative analysis by ICP-OES of the initial powder used in this study showed that the manganese content in the raw powders was less than 1 ppm, being insufficient to give a blue color. The color change observed in the presintered sample may be due to the formation of oxygen vacancies and change in the depth of bandgap.

**Figure 7:**
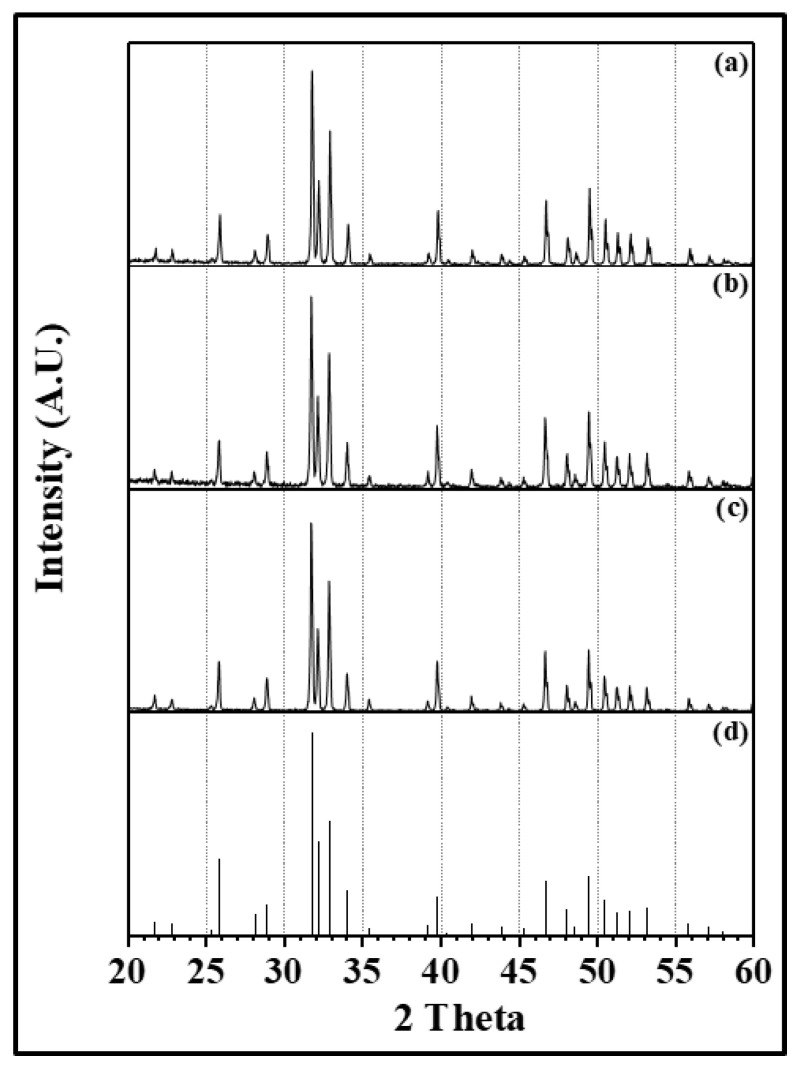
XRD patterns obtained from (**a**) the part of the flash sintered sample with no whitening, (**b**) the whitened part of the flash sintered sample under 1200 V, (**c**) the pale blue color sample after normal sintering at 1200°C for 5 minutes, and (**d**) the PDF file (No. 9-432) of hydroxyapatite.

The whitening started at the anode and proceeded toward the cathode. The times spent for whitening samples were the same regardless of the presintering condition or applied voltage. It is reasoned that the whitening was nothing to do with a flash phenomenon. During whitening, neither the electric current nor the temperature of the sample surface did not increase. When the sample was cooled before the completion of whitening, one could observe the front line of whitening on the sample surface. XRD analysis of the sample with whitening (Figure 7) showed barely any phase other than the hydroxyapatite was detected, implying that the color change is not caused by phase transformation. Blue color of the presintered sample is a result of formation of oxygen defects. When an electric field is applied at high temperature, the whitening occurs because of change in oxygen defect concentration and band gap levels.

## 4. Conclusions

The onset temperature of flash occurrence during flash sintering of hydroxyapatite sample under various processing conditions was investigated. Flash occurred at a lower temperature by 50–100 °C in vacuum than in air because of the higher defect concentration. Under the elevating temperature condition, multiple flashes occurred due to the increase in defect concentration with temperature. The thicker the sample, the lower the flash onset temperature. It was because of the decrease in specific surface area and the decrease in the amount of heat loss. Flash occurred not only in the green sample but also in the presintered samples having a density of 86%–100% and a grain size of 0.2–0.9 μm. It was observed that the higher and larger the density and grain size of the sample, the higher the flash onset temperature. This was because electrical conduction through the grain boundary and the surface of the pores was suppressed due to decrease of the fraction of grain boundary and the surface area with an increase of density and grain size. When the sample was presintered in air without applying an electric field, its color changed from white to a pale blue. When the presintered sample was flash sintered by applying an electric field, the color changed back to white. However, the color change was not a part or prerequisite of the flash sintering process.

## Data Availability

The data can be requested from the corresponding author.
